# Effects of silver nano-particles on sperm parameters, number of Leydig cells and sex hormones in rats

**Published:** 2014-02

**Authors:** Mohammad Ebrahim Baki, Sayyed Mohsen Miresmaili, Majid Pourentezari, Esmail Amraii, Vahid Yousefi, Hamid Reza Spenani, Ali Reza Talebi, Morteza Anvari, Mohammad Fazilati, Ali Asghar Fallah, Esmat Mangoli

**Affiliations:** 1 *Department of Biology, Payame Noor University, Isfahan, Iran.*; 2 *Science Faculty, Science and Art University, Yazd, Iran.*; 3 *Science Faculty, University of Sistan and Baloochestan (usb), Zahedan, Iran.*; 4 *Department of Biology and Anatomy, Shahid Sadoughi University of Medical Sciences, Yazd, Iran.*; 5 *Research and Clinical Center for Infertility, Shahid Sadoughi University of Medical Sciences, Yazd, Iran.*

**Keywords:** *Silver nano-particles*, *Sperm parameters*, *Sex hormones*, *Leydig cells*

## Abstract

**Background:** Nano-particles are extensively employed in most industries. Several studies have been started to explore the probable detrimental effects of nano-particles on human reproduction. However, there is insufficient and controversially evident of effects of silver nano-particles on sperm parameters and other reproductive indices.

**Objective: **Investigation of the effects of silver nano-particles on sperm parameters, sex hormones and Leydig cells in rat as an experimental model.

**Materials and Methods:** In this experimental study, 75 male prepubertal Wistar rats were categorized in five groups including control group and 4 experimental groups (n=15 in each group). The rats in the experimental groups were fed silver nano-particles (60 nm in dimension) with concentrations of 25, 50, 100, and 200 mg/kg/day. After 45 days (about one duration of spermatogenesis in rat), samples of blood were taken from the rats for testosterone, leuteinizing hormone (LH), and follicle stimulating hormone (FSH) assessments. Afterwards, the epididymis and the testis of each rat were dissected for analyzing sperm parameters and Leydig cells.

**Results:** The results demonstrated a statistically significant reduction in number of Leydig cells in experimental groups compared to control one. In addition, the data showed a reduction in testosterone and a rise in LH level which was more obvious in high doses (p<0.05); however, FSH level showed a reduction but it was not statistically significant. A significant decrease was also found in sperm motility and normal sperm morphology in the experimental groups compared to the control one.

**Conclusion:** Our results demonstrated that silver nano-particles, in addition to interruption in functions of sex hormones, can diminish the number of Leydig cells and sperm parameter indices. It should be noted that the effects of nano-particles on reproductive indices are dose-dependent.

## Introduction

The term "nano-technology", which originally was used by Norio Taniguchi, is the technology of production of materials with nano-meter dimensions (1). According to the definition used by the scientific communities relating to nano-technology, a nano-particle is defined as a particle with dimensions between 1 and 100 nano-meters (2). The scale of materials and structures used by nano-technology is the principal distinguishing difference between nano-technology and the other technologies. Immediate effect, greater stability, and antimicrobial properties are among other important and special characteristics of silver nano-particles which cause these nano-particles to be widely used in most fields, including foods production, pharmaceuticals, medical diagnoses, biotechnology, electronics, computer and other scientific fields (3).

It should be noted that the effects of these particles on cells and organs and also the interaction processes between these particles and cells/organs are not yet understood. So far, many attempts have been devoted to show the desirable characteristics of silver nano-particles in medicine; nevertheless, few substantial efforts have, in comparison, been exerted to assess the undesirable or deleterious effects of the nano-particles. Recently, considerable adverse effects of such materials on human and the environmental health have been reported. It is demonstrated that some nano-particles can produce reactive oxygen species (ROS) which cause toxicity in the laboratory environment (4, 5). Nano-particles can even easily pass through cell membranes, the blood-brain barrier, and blood-testis barrier (6, 7).

Studies demonstrate that prolonged exposure to colloidal silver or silver-salt precipitates can cause dermatological conditions, such as argyriaandargyrosis (8). Also, animal research suggest that inhalation, indigestion or injection of nano-particles can lead to the sedimentation of them in skin or lungs and their later movement from the primary sedimentation sites to secondary sites such as liver, spleen, kidneys, muscles, brain, ovaries, and testes (9, 10). Due to insufficient and controversially evident of effects of silver nano-particles on male reproductive performances, the present study was designed to investigate the impact of silver nano-particles (Ag-Nps) concentrations on sperm parameters, number of Leydig cells and sex hormones profile in Wistar rats as an experimental model.

## Materials and methods

**Animals and treatments**

The size of synthesized silver nano-particles was 70 nm (Cat. No. D-12486 Plasmachem gmbh, Berlin, Germany). In this experimental study, 75 male Wistar rats were purchased from animal house of Yazd Infertility Center and caged in light controlled room, and were fed rat chow with access to water ad libidum for 2 weeks before experiments. 

The animals were divided into five groups (n=15 in each). Control animals (group A) didn’t receive any silver nano-particles during study. In the first experimental group (B), the animals were fed by silver nano-particles at a concentration of 25 mg/kg. The second group of animals (C) received silver nano-particles at a concentration of 50 mg/kg orally. The 3^rd^ experimental group (D) was fed silver nano-particles at a concentration of 100 mg/kg. Finally, the 4^th^ experimental Group (E) recieved silver nano-particles at a concentration of 200 mg/kg. 

Each experimental group was regularly fed by silver nano-particles at above mentioned concentration for 45 days (about one duration of spermatogenesis) at 12-hour intervals using oral gavage. This experimental prospective study was approved by the ethics committee of Research and Clinical Center for Infertility, Shahid Sadoughi University of Medical Sciences, Yazd, Iran.

**Blood sampling and hormonal assay**

After completion of the period, the rats were put under anesthetic and 3-4 ml blood samples were taken from their ocular veins. To collect blood serum, each sample was gently tipped in a clean test tube and allowed to remain still for about 15-20 min at laboratory temperature. Afterwards, the test tubes were spun at 3000 rpm for 10 minutes and, by the use of a sampler, blood serum at the top was carefully separated. Finally, the sex hormones were measured by Eliza technique. 

**Leydig cells counting**

The Leydig cells counting was done by a method which was described elsewhere (11). In summary, each rat was anesthetized with chloroform and then killed by cervical dislocation and the abdominal area was sterilized with 70% ethanol. Afterwards, fat layers were removed and the testes were removed. The testes were weighed and the tissue fixation (by Bouin’s solution) and processing was done. The paraffin embedded blocks were sectioned in 4 microns and finally on a rotary microtome, mounted on slides and stained with hematoxylin-eosin (H&E) and examined under a light microscope (Ziess, Germany) (11). Differential cell counts were gathered from every 20th section to provide a 5% sample selection per testis. Histomorphometry was conducted by counting of at least 10 seminiferous tubules in each slide and the mean number of different cell types in one tubule in the control and treated groups was reported (12).

**Epididymal sperm parameters**

After 45 days, a small part of the cauda epididymis of each animal was dissected and located in 1 mL of pre-warmed Hams F10 medium (37^o^C, 5% CO_2_). Gentle tearing of the tissue was done to make spermatozoa swim out into the culture medium. The dishes were placed in the incubator for 15 min (13). Assessment of sperm motility was done according to WHO protocol (14). In brief, 10 μl of the sperm suspension was placed on a microscopic slide and coverslip. 

A minimum of five microscopic fields were assessed to evaluate sperm motility on at least 200 sperm for each specimen. The percentage of each category of sperm motility was analyzed and then was reported as follow; Rapid motility (Grade a), Slow motility (Grade b), Non progressive motility (Grade c) and Immotile sperm (Grade d). For evaluation of sperm morphological anomalies, a drop of sperm suspension was smeared onto a clean glassy slide. The smear was then air dried and fixed in a mixture of equal parts of ethanol and ether. 

The slides were stained with Papanicolaou stain. Dried stained slides were scanned under oil immersion (X100 objectives) for morphological abnormalities. A total of 200 spermatozoa per sample were classified according to their morphology; such as normal, coiled mid piece, hair pin (a kink at the annulus, usually 180), bent tail (a kink at the annulus, usually 90), coiled tail, double head, amorphous head, triangular head, pin head and cytoplasmic droplet. In each sample, the sum of abnormal spermatozoa was expressed as percentage (15).

**Statistical analysis**

Statistical analysis was performed by SPSS 18 software for Windows (SPSS Inc., Chicago, IL, USA). ANOVA test was applied to compare the data between groups and the term ‘statistically significant’ was used to signify p<0.05. All data were expressed in mean±SD.

## Results

**The number of Leydig cells**

Comparison between tissue sections and counting of cells indicated that there was a significant reduction in the number of Leydig cells in the experimental groups (p=0.001), and this was especially prominent in higher concentrations (100 mg/kg and 200 mg/kg) (Table I).

**Serum FSH**

Evaluation of the blood serum follicle-stimulating hormone (FSH) analysis (Table II) revealed that in comparison to the control group, the concentration of FSH in the experimental groups, had a reduction but it was not significant (p=0.210).

**Serum LH**

Evaluation of the blood serum leuteinizing hormone (LH) analysis revealed that the concentration of LH had a significant rise (p=0.002) in the experimental groups (Table II). This rising was related to increase in the dosage and in experimental group 4 it was at the maximum level. 

**Serum testosterone**

Evaluation of the blood serum testosterone analysis (Table II), revealed that the concentration of testosterone in experimental groups had a significant reduction when compared with control group (p=0.000). 

**Sperm parameters**

The results indicated a significant reduction (p=0.002) in sperm progressive motility (Grade a and b) which was related to dose of nano-particle uptake (Table III). The decrease in sperm motility was more prominent in the concentration of 100 and 200 mg/kg. In experimental groups, a significant increase was found in spermatozoa with non-progressive motility (Grade c), and immotile spermatozoa (Grade d), when compared with control group (table III). 

The results of the different concentrations of silver nano-particles on sperm morphology indicated a significant reduction (p=0.02) in percentage of normal spermatozoa in the experimental groups when compared with control one (Table III). It should be noted that the reduction was related to the dose of nano-particles.

**Table I:** Comparing the number of Leydig cells in experimental and control groups

**Groups**	**Number of Leydig cells in each field (** **mean±SD)**
Control (A)	22 ± 3.39
Expremental (B) 25 mg/kg	21.4 ± 2.30*
Expremental (C) 50 mg/kg	20.4 ± 2.30*
Expremental (D) 100 mg/kg	19.8 ± 0.83*
Expremental (E) 200 mg/kg	18.4 ± 0.54*

* Statistically significant difference (p<0.05) between experimental and control groups

**Table II:** Concentration of FSH, LH and testosterone in different groups

**Groups**	**FSH concentration** **(MIU/ml) (mean ± SD)**	**LH concentration** **(MIU/ml) (mean ± SD)**	**Testosterone concentration** **(mg/ml)** ** (** **mean ± SD)**
Control (A)	0.23 ± 0.13	0.19 ± 0.08	4.70 ± 1.39
Expremental (B) 25 mg/kg	0.22 ± 0.10	0.19 ± 0.07	4.70 ± 1.30
Expremental (C) 50 mg/kg	0.19 ± 0.07	0.22 ± 0.12*	3.80 ± 1.64*
Expremental (D) 100 mg/kg	0.21 ± 0.13	0.24 ± 0.05*	3.10 ± 0.74*
Expremental (E) 200 mg/kg	0.20 ± 0.10	0.37 ± 0.05*	2.30 ± 0.83*

* Statistically significant difference (p<0.05) between experimental and control groups.

**Table III:** The effect of silver nano-particles on sperm motility and morphology

**Groups**	**Rapid motility** **(%) (Grade a)**	**Slow motility** **(%) (Grade b)**	**Non-progressive motility** **(%) (Grade c)**	**Immotile sperm** **(%) (Grade d)**	**Rates of normal sperm morphology (%)**
Control (A)	32 ± 3.65	32 ± 3.65	24.25 ± 5.12	10.75 ± 2.98	76.20 ± 7.22
Expremental (B) 25 mg/kg	28.5 ± 3.10*	28.5 ± 3.10 *	28 ± 6.05 *	12.5 ± 2.08 *	71.60 ± 5.94 *
Expremental (C) 50 mg/kg	21.5 ± 2.64 *	21.5 ± 2.64 *	40.25 ± 2.98 *	15.75 ± 2.75 *	64 ± 4.74 *
Expremental (D) 100 mg/kg	20.75 ± 3.59 *	20.75 ± 3.59 *	37.25 ± 6.39 *	18.25 ± 3.30 *	63 ± 4.69 *
Expremental (E) 200 mg/kg	13.5 ± 5.19 *	13.5 ± 5.19 *	41 ± 3.12 *	31 ± 7.78 *	51 ± 6.20 *

* Statistically significant difference (p<0.05) between experimental and control groups. All data were expressed in mean±SD.

## Discussion

The use of silver nano-particles for various purposes has increased recently. Particles which are absorbed from the lungs, gastrointestinal tract, and skin (16) can transfer to other locations such as liver, spleen, brain and testes by blood circulation (9, 17). Several researches have shown that these particles are capable of crossing cell membranes and cause cell damage (18, 19). The results of our study showed a significant reduction of sperm parameters in nano-particle-treated animals. The decrease in sperm motility was done probably due to the influence of silver nano-particles on mitochondrial function. The effects of nano-particles on mitochondrial function of C18-4 cells had been shown by Braydich *et al* (20). They showed that silver and aluminum nano-particles are able to cross the membrane and be connected to mitochondria and acrosome of sperm. 

It is demonstrated that nano-particles can cause inflammation of the epididymis, which has a role in reduction of sperm motility (21). On the other hand, the nano-particles increase free radicals (ROS) in the cell, which can damage the sperm membrane and flagellum structure and disrupt sperm motility and morphology (4). Free radicals lead to peroxidation of phospholipids in the mitochondria of the spermatozoa and thus impair their ultimate motility (22). It is also demonstrated that ROS level is positively correlated with the proportion of sperm with amorphous heads, damaged acrosomes, midpiece defects, cytoplasmic droplets and tail defects (23).

In our study, nano-particle-treated rats had a significant decrease in sperm normal morphology when compared to control animals. It should be considered that this reduction was totally dependent on dose of silver nano-particles. In agreement to our results; Nel *et al* showed that silver nano-particles can cause the inflammation and oxidative damage. They also showed that these nano-particles may increase the rates of abnormalities in sperm morphology and genetic mutations (24). The reduction of Leydig cells in the experimental groups is logical because we had a significant decrease in plasma testosterone levels. 

Silver nano-particles like other nano-particles can get involved in the destruction of the DNA of Leydig cells and apoptosis of these cells (25). In addition, most nano-particles cause an increase in ROS levels such as superoxides and also an increase in oxidation of molecules like proteins or even DNA that leads to a reduction in Leydig cells and synthesis of testosterone (4). Some studies have indicated that nano-particles can influence Steroidogenic Acute Regulatory protein (STAR) expression (26). This protein is a transmit protein that regulates cholesterol transfer into the inner mitochondrial membrane and enhances the production of steroid hormones (27). It is possible that silver nano-particles prevent cholesterol transfer into the inner mitochondrial membrane thorough reducing StAR protein expression and eventually stop the conversion of cholesterol to pregnenolone; and as a result, the testosterone levels will be decreased. 

Regard to LH levels, our results indicated a significant rise in this hormone which was dose-dependent (maximum at concentration of 200 mg/kg). This elevation can also be the result of decrease in testosterone level. In other words, the decrease in testosterone can influence the hypothalamus in the form of negative feed-back and increase in LHRH and, as a result, in LH secretion. Furthermore, as it was mentioned before, silver nano-particles, like the other nano-particles, cause an increase in nitric oxide products (28) and as a result, an increase in cGMP level. The cGMP can raise PKG (Protein Kinase G) and such a rise enhances secretion of LHRH from hypothalamic axon terminals which finally causes LH secretion (29, 30).

We showed that the reduction of FSH level in the experimental groups was minor and insignificant (p<0.05). However, this reduction cannot be related to GnRH, because the LH has increased. This reduction may be a result of release of inhibin from sertoli cells.

## Conclusion

We showed that different doses of silver nano-particles had deleterious effects on sperm normal morphology and motility. On the other hand, these nano-particles, through influencing Leydig cells, caused reduction in testosterone levels; however, they raise LH and had minimal effects on FSH. It should be noted that high doses of silver nano-particles can affect spermatogenesis and sex hormones levels and influence fertility potential of spermatozoa in rat.
